# Towards trust-based governance of health data research

**DOI:** 10.1007/s11019-022-10134-8

**Published:** 2023-01-12

**Authors:** Marieke A. R. Bak, M. Corrette Ploem, Hanno L. Tan, M. T. Blom, Dick L. Willems

**Affiliations:** 1 Department of Ethics, Law and Humanities, Amsterdam UMC (Location AMC), University of Amsterdam, Meibergdreef 15, 1105 AZ Amsterdam, The Netherlands; 2 Department of Cardiology, Amsterdam UMC (Location AMC), University of Amsterdam, Amsterdam, The Netherlands; 3 Netherlands Heart Institute, Utrecht, The Netherlands

**Keywords:** Big data, GDPR, Governance, Research ethics, Privacy, Solidarity, Trust, Data sharing, ESCAPE-NET

## Abstract

Developments in medical big data analytics may bring societal benefits but are also challenging privacy and other ethical values. At the same time, an overly restrictive data protection regime can form a serious threat to valuable observational studies. Discussions about whether data privacy or data solidarity should be the foundational value of research policies, have remained unresolved. We add to this debate with an empirically informed ethical analysis. First, experiences with the implementation of the General Data Protection Regulation (GDPR) within a European research consortium demonstrate a gap between the aims of the regulation and its effects in practice. Namely, strictly formalised data protection requirements may cause routinisation among researchers instead of substantive ethical reflection, and may crowd out trust between actors in the health data research ecosystem; while harmonisation across Europe and data sharing between countries is hampered by different interpretations of the law, which partly stem from different views about ethical values. Then, building on these observations, we use theory to argue that the concept of trust provides an escape from the privacy-solidarity debate. Lastly, the paper details three aspects of trust that can help to create a responsible research environment and to mitigate the encountered challenges: trust as multi-agent concept; trust as a rational and democratic value; and trust as method for priority setting. Mutual cooperation in research—among researchers and with data subjects—is grounded in trust, which should be more explicitly recognised in the governance of health data research.

## Introduction

With the rise of computerised databases, privacy in relation to information technology has been a subject of societal debate for about half a century now. In medicine, a duty of confidentiality exists to safeguard access to health care and to protect individual patients’ privacy. The concept of privacy is a social construction and difficult to define: no single objective or judicial definition may suffice to describe the lived experiences of privacy across contexts (Sharon 2017; Igo 2018). Most authors agree, however, that we can distinguish between physical (bodily seclusion), proprietary (things like identity and name), and informational (personal data) privacy (Allen 1999). The latter is our concern in this article. Informational privacy in health care and research is currently being challenged by the increased globalization that stimulates information sharing and produces a growing number of international research consortia, as well as by technological developments like *big data* and *machine learning* that are known to exacerbate existing privacy risks and to create new ones (Raghupathi and Raghupathi 2014; Mittelstadt and Floridi 2016).

Namely, the era of big data enables a realisation of personalised medicine that uses networked resources to combine all kinds of information (e.g. health records, biospecimen, socio-economic and behavioural data) in order to tailor prevention and treatment to the individual patient (Prainsack and Buyx 2016). These linkages of data and the scale of aggregation create the potential for misuses and discrimination, including in terms of state surveillance or of companies denying insurance coverage based on risk profiles (Christiaans 2010; Mohammed et al. 2017). Some scholars have suggested that we are moving towards an “informational panopticon”, reflecting Jeremy Bentham’s idea of the panoptic prison where prisoners can be unknowingly observed at all times (Reiman 1995). Others believe instead that personal data are becoming “overprotected” in response to growing privacy concerns. Recently, a number of scientists have argued that the new data protection legislation in the European Union (EU) harms the public’s well-being by hampering progress in health data research (Al-Shahi and Warlow 2000; Gostin et al., 2009; Anonymous 2015; Peloquin et al. 2020).

Should we let informational self-determination prevail over data sharing for societal health benefits or vice versa? In this article we suggest an alternative way out of the dilemma: one of trust. The concept of trust stands at the core of health data research but lacks a philosophical underpinning in this context. The approach employed in this study is grounded in empirical ethics, which combines philosophy with empirical research (Kon 2009; Pols 2015). Drawing from experience within a European research consortium and from interviews with health data researchers, we describe the limits of data protection legislation and propose a new theoretical framework for data governance which is grounded in trust.

The structure of the paper is as follows. First, we sketch the current status of the ethical debate on health data research, including the legal background. Second, we reflect on data governance practice by comparing researchers’ experiences with the aims of the EU General Data Protection Regulation (GDPR) as this regulation is among the primary guiding documents for the governance of health data research. Third, we argue for a re-appreciation of trust instead of a polarized debate on privacy and solidarity. Fourth, we propose three characteristics of trust that can be utilised by researchers and policymakers to promote responsible health data research and to mitigate the barriers posed by current ethical and legal frameworks. The paper concludes with suggestions for further study.

## Ethical and legal background on the governance of health data research

The term governance has the same origin as the prefix ‘cyber’: both stem from the Greek word *κυβερνήτης* (kybernetes, translation: steersperson of a ship) that was first used by Plato to describe a person governing a state (transl. Lee 2007). Data governance refers to the making of arrangements for responsible collection, storage, usage and sharing of personal data and is needed to account for ethical concerns arising from the use of health-related data, especially when collaborating in large-scale research projects (Budin-Ljøsne et al. 2014). Governance of data processing for health research has become more important in recent decades as researchers gather data from many sources to create clinical, genetic and socio-economic profiles of data subjects.

These growing technological possibilities for big data analytics and the corresponding potential for misuses, have led to a heightened sensitivity for privacy concerns rooted in individual autonomy. At the same time, technology can make health promotion easier to realize: it supplies new ‘cans’ which result in new ‘oughts’. The can of big data may create a new ought of solidarity in health data sharing, or even a duty to participate in health data research. We discuss these two opposing ethical perspectives hereafter, before linking the debate to the EU legal framework.

### The privacy versus solidarity debate

The trend of informational privacy being viewed as increasingly important can be put in instrumental terms of preventing harms to data subjects and in principled terms of respecting subjects’ autonomy and human dignity (Bloustein 1964). Privacy can be defined in many different ways^1^ and its definition is complicated by the different nuances across country contexts. For instance, the United States traditionally have a conception of privacy grounded in liberty and freedom from the state, whereas Europeans base privacy on dignity and control of one’s public image (Whitman 2003). While there are national and cultural differences also in research ethics approaches (Gaille and Horn 2016a), the general tendency since the Nuremberg trials has been to increasingly view the autonomy of research subjects as the most fundamental value in research ethics (Pellegrino and Thomasma 1987; Wolpe 1998). Increased attention for informational privacy follows from more recent controversies in health data research as well, such as the issues around informed consent during the creation of national health databases in the United Kingdom and Iceland, and the rise of research partnerships where personal data are shared with large internet companies such as Google DeepMind (Winickoff 2006; McCartney 2014; Vayena and Blassime 2017; Horn and Kerasidou 2020).

Large-scale production of information can increase the risk of re-identification and may lead to “function creep”, i.e. using the data for purposes not originally specified. Anonymizing data is sometimes seen as the solution, but anonymization can lead to unreliable results while it may not even suffice (both technically and conceptually) to protect people’s privacy in our increasingly networked society (Barocas and Nissenbaum 2014; Andersen and Storm 2015). As a result of these new information technologies that enable data mining, our conceptualisation of privacy is changing (Kamphof 2017). Namely, recognizing the limits of anonymization, privacy is increasingly conceptualised as *control*. This is represented in formalised informed consent procedures and data access requirements (consider GDPR Recital 7: “Natural persons should have control of their own personal data”).

While measures of control may be necessary, they are never sufficient. It has been argued many times over that the burden of privacy should not be borne by individual data subjects, especially given the well-documented lack in understanding of their consent among people who donate data (Eisenhauer et al. 2019) and because privacy is about more than being able to say ‘yes’ or ‘no’. Privacy is also about how the data are used and by whom (Andrew and Baker 2021). Accordingly, Bredenoord and colleagues criticise the ‘consent or anonymise’ approach and propose that we best protect people who donate data or tissue by reframing informed consent in terms of ‘consent for governance’, i.e. focused on research infrastructure rather than on study content (Mostert et al. 2016; Boers and Bredenoord et al. 2018). While this is arguably what many research projects do already, the approach highlights well the limits of relying on consent alone and shows that health data research *always* requires a protective layer of sharing agreements, ICT security, and potentially oversight by research ethics committees (Ploem 2006). The question is whether such a protective layer would be sufficient and abolishes the need for informed consent, in favour of societal health benefits, as some argue.

Namely, in response to legislative burdens of data protection, debates have started on whether *data solidarity* (i.e., supporting the health of future others by sharing one’s personal data) rather than privacy would be the proper basis of health data research (Prainsack and Buyx 2016). Proponents of this argument suggest that a ‘neoliberal’ focus on autonomy undermines social institutions, that the harm due to non-use of health data can be greater than harm from uses (Jones et al. 2017), and that minimal risk research should not require consent (Mann et al. 2016). They think that the possibilities that big data analytics provide, create an ‘ought’ for data sharing. In response to increasing individual freedoms and a declining feeling of community, a push for data solidarity mirrors what is arguably a ‘communitarian turn’ in bioethics (Chadwick 2011; Ogunrin et al. 2018).

We have seen this in the response to the COVID-19 pandemic: when humans are suffering as a global sick body, some political actors think this creates priority for mass surveillance over individual privacy (Couch et al. 2020).^2^ Political mentions of data solidarity were found across Europe already in the pre-pandemic era. One example is a letter to parliament by the then Minister for Medical Care in the Netherlands who characterised data as “the new social revolution” and argued that since the cost of the Dutch healthcare system is shared by all citizens, regardless of whether they need it, the same principle should be envisaged for data (Bruins 2018, p. 10). In response, Dutch ethicists commented that solidarity is not without risks as personal data sharing limits self-determination and can contribute, for instance, to profiling based on lifestyle (Niezen et al. 2019).

It thus seems that prioritizing data sharing and solidarity over individual data privacy, as well as the other way around, involves important trade-offs that prevent achieving a general consensus in this debate. Further on in this article we will propose an alternative framing for the governance of health data research, namely one of trust, which could help to get out of this standstill.

### General data protection regulation of the EU

Just as information technology develops over time, legal documents are not set in stone. The increasing technological possibilities and international collaboration are reflected in the development of data protection legislation such as the GDPR which came into force in May 2018. In contrast with the 1995 Data Protection Directive, the new law is directly applicable in all EU Member States and applies to all EU citizens, no matter their location. It also updates the Directive by explicitly mentioning genetic data, and has a stronger focus on accountability and high fines for data breaches (for an overview of relevant changes, see Bak et al. 2018). Researchers often highlight the changes caused by the GDPR and sometimes fail to see that specific principles and requirements were already included in the earlier Directive. For instance, anonymization requirements have not changed: the new law only clarifies that pseudonymised data is still considered personal data.

Along with national laws that specifically govern the health care sphere (e.g. rules about medical confidentiality), the GDPR aims to protect data privacy through various principles and through practical requirements such as the mandatory conducting of a Data Protection Impact Assessment (DPIA) for large-scale health datasets. It is based on two legal rights that overlap: namely, the right to data protection which is grounded in the broader right to privacy. The idea behind the regulation is that harmonisation across the EU may be more effectively pursued if data protection legislation comes in the form of a regulation that applies directly in all countries, in contrast with the former directive. As stated in the law’s explanatory recitals, the GDPR was created to establish a higher level of privacy protection within a more harmonised European framework:

Those developments [technological advances and globalization] require a *strong* and *more coherent* data protection framework in the Union, backed by strong enforcement, given the importance of creating the *trust* that will allow the digital economy to develop across the internal market. (Recital 7 GDPR, emphases added)

This excerpt shows the GDPR’s dual aim of a strong and more coherent framework: i.e., better protecting personal data privacy *and* harmonising the legal framework to support data sharing. It also shows that these aims of the new data protection framework relate to a broader imperative of creating trust of data subjects (people whose data is used or ‘processed’) and of data processors and controllers. In what follows, we describe our experiences in a European health data research consortium to investigate how these aims of the GDPR play out in practice and what this entails for the privacy-solidarity debate. We acknowledge the complex juridical reality in which national (health) law also plays a role but we use the GDPR’s aims as a framework for highlighting issues related to health data research governance.

## Challenges for health data research under the GDPR: experiences of the ESCAPE-NET consortium

Our observations about the governance of health data research are drawn from the authors’ experiences in an international research consortium called ESCAPE-NET (the European Sudden Cardiac Arrest network towards Prevention, Education, New Effective Treatment). This EU Horizon-2020 funded research consortium is building a large database of sudden cardiac arrest (SCA) patients for observational studies aimed at improving SCA prevention and treatment (Empana et al. 2018). Approximately one-fifth of all deaths in Europe are caused by SCA, a condition which is lethal within minutes if left untreated, and survival rates vary between 5 and 20% (Tan et al. 2018). Because a combination of multiple factors can cause SCA and treatments differ between European geographies, large datasets and international collaboration are needed. ESCAPE-NET combines data from SCA cohorts (~ 85,000 people), genetic studies (~ 15,000 samples) and prospective population cohorts (~ 55,000 people) into one harmonised database. Individual datasets may include clinical information collected from hospitals, emergency medical services (EMS), general practitioners and patient surveys, as well as pharmacological, socio-economic and genetic information.

During the course of the project, the authors conducted on-site observations and held interviews with health data researchers from ten research groups that contribute patient cohort data to ESCAPE-NET (Fylan 2005). Qualitative semi-structured interviews were conducted with 16 ESCAPE-NET researchers between May and September 2018, around the time of introduction of the GDPR, while observations were done over a three-year period. The interviewed research groups were spread across six European countries (NL, IT, FR, DK, SE, CZ) and there was variation in the types of cohort studies performed (e.g., with or without DNA collection). Moreover, the authors participated in consortium meetings and expert conferences and were involved in the ethico-legal approval processes for ESCAPE-NET. Here, interview findings will not be systematically presented but rather used to illustrate the governance issues encountered.^3^ In our theoretical analysis, we draw mainly on phenomenology to reflect on concepts arising from ESCAPE-NET researchers’ and our own experiences (Aspers 2009; Saraga et al. 2019). We encountered three (potentially) negative effects of the GDPR in the practice of health data research and describe hereafter how these experiences conflict with the aims of the regulation.

### Data protection without reflection

The first aim of the GDPR is to better protect personal data in an increasingly digital and globalised society. During the GDPR implementation phase, the ESCAPE-NET project’s focus was on obtaining approvals from Research Ethics Committee or Institutional Review Boards (RECs/IRBs), devising data processing and transfer contracts, and sorting out legal questions such as in which country to host the server for the database—which was finally done in Denmark because it had the most stringent requirements about data leaving the country. Addressing legal challenges was found to be costly in terms of time, money and workload. In eight of ten research groups, researchers expressed that the introduction of the GDPR hampered their research.^4^ This burden seemed to decrease when institutions created or updated standard templates (e.g., for data transfer agreements and DPIAs) and as legal advisers became more familiar with the new European GDPR framework. Some costs for the researcher inevitably will remain and this seems acceptable given the importance of protecting patients’ fundamental data protection rights.

However, while interviewed researchers agreed in theory with the stronger protections afforded by the GDPR, they felt that data protection increasingly comes down to “checking boxes” and using the correct phrasing. Indeed, studies have shown that the effectiveness of DPIAs “varies depending on whether there is in-house privacy expertise [and that] more often than not, they are compliance checks completed without a broader analysis of privacy risks” (Bayley et al., 2007). Before the law came into force, one ESCAPE-NET investigator said he thought that registry research would become easier because the general public would be made more aware of researchers’ responsibilities for proper data protection. However, in practice the stronger requirements may not provide practical tools for data protection nor support reflection on underlying values. Another researcher expressed his frustration as follows:

Principal investigator: “This is eating up so much of people's time, and I am really bothered about this, because we spend less and less time on research and more and more time on *doing the right wordings in the approvals*. And if the EU or the government really wants us to continue to do research on such high level, they should really think about how to make it easy and not to... I mean now it is almost like they are not our friends.”

When data governance is framed merely in terms of compliance with legal and ethical requirements, a risk of *routinisation* ensues. Ploug and Holm (2013) introduced this concept to describe the phenomenon where research participants are asked repeatedly for informed consent, and as a result providing consent becomes an act of routine without reflection. Informed consent then loses its function of protecting autonomy. Similarly, we note that the focus on safeguards and checklists can also cause routinisation among *researchers* trying to practice good data governance. One might argue in an Aristotelian manner that routinisation could stimulate good governance: namely, by cultivating virtue through creating habit and practice among researchers following the data protection procedures (Jonas 2018). This may be true in simple situations but for more complex research projects working with sensitive health data, we find that stimulating checkbox routine without further reflection can frustrate the underlying moral values of data protection tools such as DPIAs.

### Destabilizing the trust relation between researchers

The ‘stronger’ data protection framework can have another secondary result: while the GDPR text mentions the importance of creating trust, such a trust relation may become destabilised by an excessive focus on legal compliance and control. Between ESCAPE-NET researchers, levels of pre-existing (‘ontic’) trust were high. For instance, when discussing whether oversight on the scientific quality of studies was needed, one of the executive committee members did not find this necessary because *“they know what research is and I trust their judgement”*. Trust makes cooperation easier as it removes incentives for monitoring (Luhman 1979). Researchers who trust each other to handle data responsibly, and who enjoy collaborating, are more likely to share data (Budin-Ljøsne et al. 2014). Indeed, in ESCAPE-NET the existing trust between scientific partners leads to solidarity in data sharing and collaborating for patients’ benefit:

Principal investigator: “It's a good group as well. You know, when you do research it's a lot about trust and that's something I think we have in this group. We know each other from previously. We know of each other's work. (…) I mean it is a question of whether they use the data correctly. Ethical and trust is a bit the same in these situations. That they use the data correctly is one thing, and of course the breach of data... If they are not secure enough. And that is difficult when you are not there, so you really need good trust.”

However, the legal and technical complexity of data protection requirements and data sharing contracts, combined with the risk of high fines, undermined collaboration between partners in the ESCAPE-NET project by complicating data sharing. We also saw that strict legal measures (related to the GDPR or to requirements for medical secrecy) led to researchers having difficulty in cooperating with external data suppliers like hospitals or ambulance services who became hesitant to share, and with RECs and DPOs who became increasingly cautious in approving research proposals. ESCAPE-NET is a relatively young project that mostly shares data within the consortium, but the legal complexities may complicate future cross-consortium collaborations, as was seen in other studies (Budin-Ljøsne et al. 2014). Moreover, we encountered interpersonal trust issues that formed between researchers within the participating research groups:

Postdoctoral researcher: “There is DNA information that is encrypted and separated from the database. I am the only one who can link it with a key. That is nice, but it is also very annoying because if we need to link with phenotypic information, then I am the only one who can do that. It takes a lot of time. I think I can delegate this, but at present I don't trust anyone enough yet to do it rightly.”

While we discovered the importance of trust within a successful collaboration like ESCAPE-NET, we found that trust between researchers is an understudied topic. Most existing literature focuses on the trust of research participants, given that public trust in science has been declining in the past decades. This lack of trust reduces research participation and negatively impacts the public’s perception of research (Kraft et al. 2018).^5^ The response to such worries about trust generally consists of increased regulation and oversight on research, including requirements of accountability and transparency, and the creation of contracts such as informed consent forms and data sharing agreements (Sheehan et al. 2020; O’Neill 2002). Wolpe (1998) has referred to these as ‘rituals of trust’ that emerge when ontic trust, in this case of the public towards research, is scarce.

The GDPR, with its codification of data protection into DPIAs and promise of stronger enforcement, may be an example of such a ritual of trust—despite the existence of research exemptions. We observed that successful implementation of formal data protection safeguards requires some existing trust but can also ‘crowd out’ this same trust between researchers. Trust in health data research is not incompatible with regulation, yet after a certain threshold the gathering of information to ensure that the other party can be trusted (e.g. by endless contact through lawyers in order to draft joint data controller agreements, as was needed in ESCAPE-NET), will destabilize the pre-existing relation of trust (Baier 1986; Dasgupta 1988). After this threshold, rituals of trust can create distrust that complicates cooperation and data sharing for the public good.^6^

### Incoherent guidance due to disagreement about ethical values

Lastly, while the second aim of the GDPR is to improve coherence, it still allows Member States their own interpretation of certain provisions including research exemptions (van Veen 2018). Some countries are more restrictive than others and this can complicate the establishment of a joint database shared between different countries (Nilstun et al. 2006; Haneef et al. 2020). For instance, the use of deceased persons’ data is not covered by the GDPR but can be regulated nationally (Bak et al. 2020): in ESCAPE-NET, some groups could not use these data, which negatively affects study validity and may result in bias. Researchers also noted that their collaboration was affected by national *and* local variation among data protection officers (DPOs) and research ethics committees (RECs/IRBs) (Vandenberghe 2019; de Lange et al. 2019). As a result of different interpretations by experts at participating institutions, a number of studies were stopped until legal questions were sorted out: this took up to two years for some groups.^7^

Differences in (interpretation of) regulation are due in part to cultural and political factors. For instance, in Scandinavian countries the importance of registry-based epidemiology is engrained in the national culture (Bauer et al. 2014). Another reason for variation is that laws are necessarily formulated in broad terms and may not apply directly to the specific context, in this case emergency medicine where prospective patient consent is impossible. One researcher summarised:

Postdoctoral researcher: “There are codes of conduct on using patient material. But they never treat my situation. They do not deal with the issues that I am facing. (…) We have an approval now from the ethics committee, but then you still have to go to the DPO and she can still say: no, this is not right.”

Several interviewees expressed a desire for more legal guidance. As Kafka wrote (1979, p. 128), “it is an extremely painful thing to be ruled by laws that oneself does not know”. A researcher present at a conference about ESCAPE-NET, noted that the insecurity of researchers themselves, who are legitimately worried about fines and about the continuation of their research, also harms research:

Researcher: “The ethics committee and data protection officers told us: the law does not keep you from doing your research. It is only your own fear and uncertainty of doing the research and taking the risk of data breaches if you don’t know what you are doing.”

However, all laws remain to a certain extent open for interpretation. A legal expert with whom we spoke about ESCAPE-NET commented on why there is so much discussion among jurists: “one might lean more towards the principle of privacy protection, whereas another might attach more value to scientific research and data sharing”. It is unclear how researchers should navigate these various interpretations of what good governance is, especially when collaborating in international consortia with involvement of many different data protection officers and legal teams.

## A proposal for trust-based governance of health data research

We have seen that the aims of the GDPR were not reflected in researchers’ experiences. The current data protection framework can have the potential negative effects of reducing data protection to checkbox exercises which promotes routinisation and crowding-out of trust, and of leading to incoherent guidance due to different interpretations. Since law can be seen partly as solidified morality, the underlying issue here is one of ethics: in their interpretation of the GDPR, those involved in health data research seem to be searching for an ethical foundation for good governance.

### One principle to rule them all?

Indeed, van Veen (2018) notes that “[legal texts] could be subsumed under informational self-determination versus solidarity” and “the future of biomedical research in Europe will be decided not only by the GDPR text but also by the outcomes of the debate on those values”. Which ethical value or principle, then, should be given priority when devising governance policies for health data research? The issues encountered by researchers in ESCAPE-NET can be traced back to the privacy-solidarity debate described in the background section of this paper. Hummel and Braun (2020) have argued, for instance, that in data-driven medicine there is a conflict between the *good* of data sharing and the *right* of addressing privacy harms, and that a balance ought to be found between solidarity and “foundational norms of justice”. As mentioned earlier, bioethicists have wide-ranging views on what would be an appropriate balance and the debate has not been concluded. We argue that it *cannot* be, if scholars continue to frame privacy and solidarity as strictly opposing values and consider one of them to be more foundational.

We find that the problem lies partly in a lack of clarity on the meaning of these two principles. Political documents generally remain vague about how privacy and solidarity are conceptualised and academia fares no better: while there is a blossoming scholarly literature on the concept of solidarity in relation to health data, there exists no consensus yet on how it should be defined, other than as something “contributing positively to the social fabric of society” (Prainscack and Buyx 2016; Dawson and Jennings 2012). Privacy and solidarity are difficult to define not only by themselves but they are also very much linked: autonomy is a relational property and can be informed by the concept of solidarity (Mackenzie and Stoljar 2000; Gaille and Horn 2016b). An autonomy-inspired striving for individual privacy paradoxically leads to more dependence on others; and individual benefits may give rise to group-level privacy harms (Van der Loo and Reijen 1993; Coughlin 2008).^8^ Thus, while the debate is often framed in terms of individual versus societal benefits, this distinction is not helpful.

Moreover, there is no objective evaluative standard for balancing these values. An appropriate shared standard may be especially difficult to find in international collaborations if partners do not share the same morality (Musschenga and Meynen 2017). What can be considered good governance, depends on *contextual* factors and there is simply no one fundamental value to ground our actions. Philosophers have long known that all rules may ground out on something arbitrary and merely stem from how we choose to organise society. As Kant (1992) said, metaphysics is an ocean without shore and lighthouse (2:66.1–6). In this ocean of uncertainty, our values are like planks of a floating raft that can only be built into a ship by standing on one of the other planks—one cannot stand outside the raft or find final principles by diving down (Neurath 1973; Lorenzen 1987); or like a wiki where all entries link to each other based on how the developers decide they should (Lynch 2016). We argue therefore that what is needed is not a search for final principles, but a re-appreciation of trust as the rope that keeps the raft together.

### Promoting the social contract for research requires a re-appreciation of trust

Kamphof (2017) frames privacy “as a gift of trust” to health care professionals and our experiences and interviews in ESCAPE-NET suggest that this is also the case for research with health data. We argue that more attention for this concept of trust is needed to fruitfully address governance issues and eliminate the privacy-solidarity dichotomy in the ethical debate on health data research. Both privacy and solidarity are in a sense ‘without ground’ and finding a good balance between them requires *trust* as the basis for the social contract between researchers and data subjects (Allen et al. 2019).

That is, trust is needed in the world as it would not be economically efficient, nor practically possible, to have everyone know and control everything that affects them (e.g. scientific knowledge is impossible without trust: we have to trust scientists’ testimony in believing that the earth is round). The commonality of rules is based on unconditional trust and trust is therefore a type of social capital that enables people to cooperate (Fukuyama 1995). William James, an early phenomenological philosopher (Edie 1970), already noted that ethics by definition involves trust in others: we cannot always wait for evidence as we might risk missing out on valuable societal truth (James 1897).

A social organism of any sort whatever, large or small, is what it is because each member proceeds to his own duty with a trust that the other members will simultaneously do theirs. Wherever a desired result is achieved by the cooperation of many independent persons, its existence as a fact is a pure consequence of the precursive faith in one another of those immediately concerned (Section IX)

Especially with the rise of big data analytics where the consequences of research and data use become even more uncertain and the collaborations more widespread, trust is important for promoting both data protection and data sharing in health research. We already noted that researchers who trust each other to handle the data responsibly, are more likely to share data (Budin-Ljøsne et al. 2014). Similarly, trust has always been characteristic for the physician–patient relation where patients enter the “sick role” exempting doctors from ordinary people’s responsibilities, and a key function of medical research ethics codes is to foster public trust (Parson, 1951). We trust doctors partly because we know they are covered by contracts, professional codes, and laws. In a study from the United States, patients’ trust in researchers was the most powerful determinant for the kind of control they desired over their medical records: when trust is low, patients desire explicit informed consent (Damschroder et al. 2007).^9^

Formalised measures may play an important role in promoting trust between parties by demonstrating reliability and reducing uncertainty, especially when societal values are in flux. For instance, legal contracts between researchers or institutions (e.g. data transfer agreements) serve as an implementation of the social contract for data science. They are what Hannah Arendt called islands of predictability: “to make a promise is to predict the future” (1978). However, like most things in life, health data research always includes some degree of risk and unpredictability. Graham et al. (2022) describe how the word trust is often misused because Trusted Research Environments or Trusted Third Parties actually *reduce* the need for trust in health data research by increasing control over the data. This fits with a change in motivation for trust in healthcare that Calnan and Rowe (2007) describe as moving “from affect based to cognition based trust”. Trust based on cognition, which involves calculation and risk analysis, is inherently based on control rather than faith. We find that this is not real trust, but merely reliance, and that trust based on affect remains necessary in an uncertain world.

Affect-based trust is reliance “plus some extra factor” (Hawley 2014; Goldberg 2020). In an exploration of trust in the context of the UK’s National Health Service, Sheehan et al. (2020) showed that this extra factor lies in the fact that trust is associated with gratitude when vindicated and with betrayal when it is not. According to Baier (1986), betrayal is the appropriate response when someone is relied on to act out of goodwill. For instance, recall how one of the ESCAPE-NET investigators said about the European Commission that it was “almost like they were not *friends* anymore”, which shows betrayed trust rather than misplaced reliance. Overly formalized data protection measures may eventually crowd out trust by mistaking it for reliance, or by focusing solely on public trust and disregarding trust between other actors in research. So how can these complex relations be addressed and trust used to promote good governance in health data research, in a way that goes beyond the polarized debate on privacy and solidarity? In the final section of this paper we make some suggestions based on our conceptualisation of trust in health data research.

## Conceptualising trust: three pragmatic aspects

In this section, we provide practical trust-based suggestions for balancing out the potentially negative impact of data protection policies. We do so by proposing a three-part conceptual framework for trust in health data research that establishes trust as: a multi-agent concept; that is rational and democratic; and that can help with priority-setting among ethical values.

### Trust as multi-agent concept

The dominant philosophical paradigm of trust is one of interpersonal trust, e.g. between doctor and patient, and trust has been defined simply as *the belief that the trustee will put the truster’s best interests first* (Williams 2007). However, this common conception of trust does not suffice for health data research which is always embedded in a social system. Complex research projects are therefore better compared to a multi-agent system (MAS) in computer science. Similar to a MAS, health data research is composed of multiple interacting intelligent agents and their environment, that must act together to solve complex problems. In our case study, we encountered many mentions of trust at different levels, between various people and organisations. This reflects what David Resnik (2018) calls a “web of trust” where trust connects all actors in the medical research enterprise (i.e. the people building the raft or wiki together), including research sponsors.

Of course, trust is also important in the relationship between participants and researchers. In our interviews, researchers believed data breaches would be harmful as they lead to a breach of trust in the research enterprise as a whole. For clinical research, trust is often quoted as people’s main reason for participation (Kass et al. 1996). Similarly, several ESCAPE-NET researchers have told us that they believe “the trust in the researcher should be enough” for people to decide to contribute data. In a study where we interviewed SCA patients who contributed to ESCAPE-NET, we found that trust was indeed one of the key factors for people when deciding to share their personal data for research (Bak et al., 2021). This trust mainly stemmed from their positive experiences with clinicians and with the medical institution conducting the research.

As such, the trust in health data researchers or appointed intermediaries like a Trusted Third Party (TTP) constitutes a kind of ‘institutionalised trust’, since the interpersonal trust stems from knowledge about how individuals in certain positions, like doctors, are supposed to act (Nooteboom 2006; Stepanikova et al. 2009). Institutionalised trust can be diminished by negative portrayals in the media – our interviewees mentioned several data breach scandals that they feared might deter people from participating in health data research. But when Brown (2009) analysed trust among gynae-oncology patients using the work of the phenomenological philosopher Alfred Schütz he found that patients, in seeking to trust, explained away any media-related fears. In Brown’s and our studies, this type of confirmation bias seemed to come from a ‘will to trust’, e.g. a will to contribute to health research in order to help future others.

In medicine, patient trust is known to increase with the number of doctor’s visits and the duration of the physician–patient relationship (Stepanikova et al. 2009). Now big data is mediating the relation between patients and medical researchers in a new way, with the ethical duties less visible due to the distant and sometimes anonymous nature of the relationship. Moreover, health data research is increasingly performed by non-clinicians like experts in machine learning or epidemiology, and generalised trust in doctors does not suffice anymore. These factors complicate the creation of trust and may reduce the public’s will to trust researchers. If trust becomes increasingly scarce, this negatively impacts study recruitment (Ford et al. 2008). Thus, when aiming to promote trust in big data studies, it is important to take into account the more distant relation with researchers and to focus not only on data subjects but on all actors in this multi-agent system, including on the interrelations between micro and macro level actors. Namely, the connectedness between interpersonal trust and system trust is what makes trust so fragile (Bratspies 2009).

The relation with RECs/IRBs is similarly one of trust, as investigators need to be able to trust that their studies are reviewed fairly and competently (which is sometimes problematic when REC/IRB members do not have expertise in big data (Ferretti et al. 2021)). Trust in regulations like the GDPR and in regulatory agencies is another important kind of trust, that can help build a more resilient society in the face of uncertainty (Bratspies 2009). As we saw in our interviews, researchers must also be able to trust *each other* to behave competently, ethically, and professionally (Whitbeck 1995). But they may also outsource some aspects to institutional actors. For instance, the quoted researcher who hesitated to give the data linkage key to a colleague, eventually instated a TTP to manage data linkage and collection of informed consent as an intermediary between research and data subject. In addition, artificial agents can also be trusted or distrusted, which was not apparent in our case study but is a point to consider when artificial intelligence and robotics become more prevalent in the healthcare setting (Glikson and Woolley 2020).

Further practice-oriented research is needed for recommendations and criteria for promoting trust and trustworthiness in each particular actor. Our preliminary suggestion concerning data researchers is that ethics education could aid them in relating data protection rules to wider values and norms (such as human rights) as a primary reminder of the societal fundament of rules, which may help prevent harmful effects of routinisation. We also suggest that specific ethical and legal support is needed for researchers to empower them in safeguarding participants’ rights, so to ensure that people’s trust is well-placed. Guidance may take the shape of codes of conduct or lay and expert advice, which calls for increased collaboration between RECs, DPOs, ICT security and legal experts, and the general public. Future work should be informed by the public policy and social psychology literature on trust in modern institutions (e.g., Nooteboom 2006), so to provide recommendations for sustaining the multitude of fleeting relations that are inherent to large-scale data-driven health research. For instance, for building trust, an amount of funding might be better spent on one long-term health data research project than on several short-term projects.

### Trust as rational and democratic

The only protection from the unknowable is the suspending of judgment (a Husserlian ‘bracketing’ of the world, if you will), but this act of trusting involves risk and constitutes at first sight an inherently irrational decision (Möllering 2001). Acts of trust may be *prima facie* irrational actions, but can in fact be highly rational, says Brown (2009) in reflecting on Kierkegaard’s idea of the ‘leap of faith’. Professionals who are friendlier or more patient are likely to deliver more positive outcomes: thus emotions of trust can constitute a rational response to unconscious ideas about correlations between the communicative signs and the motives of the trustee. Rationality is often mistakenly equated with certainty. By drawing on previous lived experiences, data subjects will not have definite predictions of the future but can know (*feel*) how to act in uncertain circumstances. And even in absence of previous experience, trusting may still be rational when aiming to minimise anxiety about uncertainty in situations of vulnerability (e.g., when assuming the aforementioned sick role in relation to healthcare professionals; and perhaps especially in relation to emergency care providers (Zaner 1991)). Health care and research function in a system of societal norms, with its contracts and safeguards, and thus make trust plausible for socially embedded agents (Hollis 1998).

To ensure that this trust is not misplaced, however, researchers should give *reasons* that serve as *trust-tags* within a particular environment or context (Lynch 2016, p40).^10^ After all, it is the human capacity for reasoning together that makes moral progress possible (Singer 1981). Neither privacy or solidarity is more rational than the other, but discussion about these principles leads to *more democratic* decision-making about health data research. French philosopher Emmanuel Levinas (1985) argued against Heidegger that ethics does not have an essence but occurs out of concern for the Other: across the hiatus of dialogue instead of in the content of discourse (“the said does not count as much as the saying itself” (p. 42)). Therefore, in order to engage in deliberation, those involved need to accept that actions are essentially unfounded but that they still stand on a shared societal normative framework, as we argued in the section "A proposal for trust-based governance of health data research". In practice this means that rather than asking people to have blind faith, health data researchers can create trust-tags by publicly explaining their policies and by providing patients and other researchers with information about data uses and oversight mechanisms (Kraft et al. 2018).

This can be done via public and patient engagement (PPE) during the planning and implementation of studies, for instance through a steering board with patient representatives (Price and Cohen 2019).^11^ In their communication efforts, researchers need not fear being transparent about risks and uncertainties, as communicating uncertainty only has a minor impact on people’s trust (van der Bles et al. 2020). Especially engagement with people who distrust researchers, can be an opportunity to make policies more trust-promoting. While researchers should be trustworthy, the research subject as truster also has a responsibility, namely to be understanding and receptive to trust-tags.^12^ It is impossible to require guarantees against all harm and “the existence of the abyss is beyond the patient’s control, but they have materials for bridging the depth of uncertainty” (Brown 2009). The truster must be content with some level of vulnerability, as we saw that an overemphasis on monitoring will crowd out trust. Further research can study how to support the public in being responsible trusters.

In addition, because data collection is always embedded within a particular culture and trust is different in different social contexts (Sheikh and Hoeyer 2018), it has been suggested that ‘ethical meta-data’ may be useful to promote trust in international studies: i.e. the addition of information to datasets about the normative context of the study, such as the consent conditions that need to be respected when data is shared with other researchers (de Vries et al. 2014; Woolley, 2017).

### Trust as method for priority-setting

If after the exchange of reasons, moral conflicts remain between key principles of biomedical ethics (autonomy, non-maleficence, beneficence and justice), the instrumental value of trust is useful for *priority-setting.* This idea has been elaborated by David Resnik (2018) who argues that in clinical research a fifth principle (‘promote trust in research involving human subjects’) can help investigators and oversight bodies to set priorities and to resolve disputes involving the interpretation of regulations (p. 105). In case of conflict, researchers ought to ask themselves how one action or another would impact on people’s trust (of note is that promoting trust is not a ‘meta-rule’ but a prima facie rule that may conflict with other principles as well). In our view, the fifth principle also applies to non-interventional health research with data. Trust helps solve the moral dilemmas inherent to data sharing (e.g. regarding privacy vs solidarity) by serving as an alternative principle or a “shared value dimension” (Stark 2020).

For instance, in the case of ESCAPE-NET, the consortium leaders are currently facing the challenge of sustaining the database after project funding ends, and are deliberating whether attracting commercial funding would be an option. In their deliberations, they could use the principle of trust as an additional aid and apply the moral test of trust (Baier 1986), asking: ‘Would patients’ trust be damaged if they found out about this practice?’. If the initiators of the failed *care.data* programme in the UK had used this principle, they might have chosen better trust-promoting ways of informing the public about (commercial) data uses and may have still been operative (Carter et al. 2015). Even for minimal-risk observational studies, asking consent from data subjects may be valuable to create trust, as it shows that researchers are transparent and that they take patients’ preferences seriously. In order to facilitate the data subject in perceiving the researcher as competent and caring, incorporating trust into decision-making thus requires good communication (Poortinga and Pidgeon 2003).

## Concluding remarks

In our experiences with the ESCAPE-NET consortium, we found that while the central aims of the GDPR are compatible with stimulating health data research, the implementation in practice can be problematic. Formalised measures like extensive DPIAs can lead to routinisation among researchers, which may cause data protection instruments to lose their protective function, although quantitative study on the effect of routinisation is needed. In addition, the lack of (inter-)national coherence in legal requirements and in interpretations by DPOs and RECs undermines the harmonization function of the GDPR and complicates data sharing (Kaye 2011). The different legal interpretations stem partly from different views on the right balance between privacy and solidarity. We bring a new perspective to this debate, suggesting that the key does not lie in recognising either privacy or solidarity as foundational, but in a re-appreciation of trust as basis for science’s social contract.

We have shown that formal privacy measures may build trust, but that overly restrictive measures destabilize the trust relation between different actors. Attention for trust has so far focused on patient and public trust, and our findings highlight the important role for trust between researchers and with funders and oversight bodies, which should not be overlooked. We have provided practical recommendations based on a three-part conceptualisation of trust that may help to frame and promote responsible governance of health data research: trust as multi-agent concept; as rational and democratic; and as a method for priority-setting. More generally, we advocate the creation of guidelines and policies (at EU- and at project-level) for promoting trust between all the different agents in the research system, which requires dialogue with these stakeholders. This may be done through ethics education, PPE or interdisciplinary expert groups (Kamphof 2017). These initiatives should be inclusive and representative and insights may be obtained from research with tissue samples or from non-medical contexts, to transpose solutions that worked in those settings (Yarborough et al. 2009).

Of note is that the practical implications of our conceptual analysis might be different in other cultural contexts. We looked at a European consortium where pre-existing trust was high: there was already a culture of trust. In contrast, in collaborations of researchers from high income countries with researchers from low and middle income countries, trust may not be sufficient given existing power asymmetries (Kerasidou 2019). Similarly, in research with people from underprivileged communities, a model of participant-researcher relations based primarily on trust might reproduce power and knowledge asymmetries, and alternative models should be sought (Ducournau and Strand 2009). Also, even between European countries there may be differences in the viability of our proposal: work by Bekker et al. (2018) shows how consensual governance regimes like the Netherlands are more likely to successfully adopt trust-based governance approaches compared to more hierarchical and centralised countries like the United Kingdom. Trust-building models, they write, require existing trust-generating institutional conditions. In absence of these conditions, trust should be developed locally and from the ground up, through face-to-face networks.

Further work needs to take into account such country differences, and view this paper as a theoretical starting point rather than as generalizable data. Additional study is also needed on the particular conditions for conducting health data research in partnership with commercial companies which may reduce public trust (Sterckx et al. 2016). For instance, commercial access could be limited to uses that promote the public interest (Horn and Kerasidou 2020). Trust has its limits and normative study would be valuable to argue where these limits should lie in health data research.

The promotion of trust also requires recognising the limitations of localized oversight in an ICT-based research world, since health data research does not follow the traditional model of “one subject, one researcher, one jurisdiction” (Woolley, 2017). Further study is needed on the desirability and potential for harmonising governance across Europe. Increased harmonization of data protection guidelines and ethical approval processes for observational studies could help to protect patients’ rights and to promote collaboration for creating larger and more valid datasets (Ludvigsson et al. 2015; de Lange et al. 2019). In order to avoid replication of review, the ethics review of observational research could be modeled after efforts to harmonise clinical trial review processes (Dove et al. 2016). Harmonization requires, however, international agreement on definitions of complex bioethical concepts such as solidarity as well as on data protection terminology such as what constitutes anonymous data (Gaille and Horn 2016a; Wallace, 2016). In addition to or instead of harmonisation, context-based policy solutions like the use of ethical meta-data when sharing datasets can help to ensure that the governance of international collaborations is based on the values of involved patients and researchers (Thorogood et al. 2015; de Vries et al. 2014).

Finally, we wish to stress that initiatives aimed at building trust should not be one-time affairs but require sustained effort and responsiveness to changes as “our dynamic society requires a dynamic morality” (Van der Burg 2003). One area where views seem to be changing is the use of deceased persons’ data for research which has been largely unregulated at international level, and it is important to investigate the moral basis and implications before any practice becomes socially embedded (Bak et al., 2020). Moral change around concepts like privacy and solidarity is induced by big data analytics, and normative frameworks may continue to be adapted with the growing use of artificial intelligence and machine learning methods in health care and research. Where these methods run into problems around the explainability of algorithmic decision-making, trust will become even more vital.

## Footnotes

1 One influential definition of informational privacy was given by Westin (1967): *“The claim of individuals, groups and institutions to determine for themselves, when, how and to what extent information about them is communicated to others”*.

2 See also: Yuval Noah Harari (20 March 2020), The world after coronavirus. Financial Times. [https://www.ft.com/content/19d90308-6858-11ea-a3c9-1fe6fedcca7].

3 Detailed interview methods and specific findings relating to the protection of SCA data in particular (e.g., on informed consent in emergency settings) are described elsewhere (REF removed for review).

4 Partly, this may be due to the fact that with GDPR implementation, data protection fines became higher and oversight stricter – requiring institutions who had not been compliant with the former Directive to bring their outdated data protection policies up to date quickly. The implementation period also made clear the advantages of having a large EU-funded consortium: smaller parties may not be able to bear the data protection costs.

5 For ESCAPE-NET, this was experienced in a minority of studied research groups that reported declining consent rates after May 2018, but we do not possess quantitative data about the effects of media-attention surrounding the GDPR on patient participation.

6 Distrust, which serves to protect from tyranny or oppression, has received little attention in the philosophical literature, possibly because it is considered less risky than misplaced trust (D’Cruz 2019). However, the harm of distrust may lie in the non-use of data which does have important societal consequences.

7 For instance, one group created 18 different types of informed consent letters for different types of patients (children, parents of deceased children, adults, legal representatives, et cetera) and based on the kinds of data collected (with or without DNA collection). These letters were revised dozens of times in response to comments by partners who contributed data (ambulance services, hospitals), the legal department, local REC, and DPOs. Only after two years of revisions to these letters, and to the DPIA and study protocol, the study received ethics approval. In addition, as research in emergency medicine is dependent on other partners in the ‘chain of care’, a data breach at one of the ambulance services temporarily halted the supply of data from that data source.

8 Adequately protecting health databases, for instance, is impossible without involving ICT security experts. Or consider the individual cardiac arrest survivor who benefits from data research when he or she receives an implantable defibrillator based on a risk prediction model; while the collective privacy of a group would be harmed if this model leads to people with overweight and obesity being excluded from defibrillator treatment for reasons of lifestyle responsibility.

9 Of note is that also the response at the other end of the spectrum, i.e., enforcing solidarity rather than privacy, will cause trust in health data research to wither (Wertheimer 2014; Ballantyne and Schaefer, 2018). Solidarity understood as a means to keep society together (‘solid’) cannot be required from the top down, but can stem only from shared trust.

10 Whether one ought to trust or not, is relative to contextual factors (Jones 1999). Similarly, the right to data protection is not absolute but context-dependent, as it “must be considered in relation to its function in society and be balanced against other fundamental rights” (GDPR Recital 4). For example, it is conceivable to tend towards solidarity for public health research and towards privacy for certain commercial uses. This balancing act is influenced by public opinion and depends on how the GDPR is interpreted by experts, policy-makers, oversight bodies and courts: the life of the law is the plaintiff (Nader 2001).

11 In ESCAPE-NET, most groups disseminated findings but did not involve patients in the design of the research. Some researchers thought PPE would be especially valuable in emergency medicine given data subjects’ greater vulnerability, and believed that PPE would improve study quality and relevance. Others did not see the need, given the lower risks associated with observational studies compared to clinical trials, or expressed concerns about the representativeness and knowledge of patient panels.

12 Trustworthiness differs from trust in that it depends on features of the trustee, as a type of moral virtue or property (Potter 2002), while the act of trusting is an attitude that depends on features of the truster.
